# Caloric restriction reduces sympathetic activity similar to beta-blockers but conveys additional mitochondrio-protective effects in aged myocardium

**DOI:** 10.1038/s41598-021-81438-7

**Published:** 2021-01-21

**Authors:** Bernd Niemann, Ling Li, Andreas Simm, Nicole Molenda, Jens Kockskämper, Andreas Boening, Susanne Rohrbach

**Affiliations:** 1 Department of Cardiac and Vascular Surgery, Justus Liebig University Giessen and University Hospital Giessen and Marburg, Giessen, Germany; 2 Institute of Physiology, Justus Liebig University Giessen, Aulweg 129, 35392 Giessen, Germany; 3 Department of Cardiac Surgery, Martin Luther University Halle-Wittenberg, Halle, Germany; 4 Centre of Medical Basic Research, Martin Luther University Halle-Wittenberg, Halle, Germany; 5 Institute of Pharmacology and Clinical Pharmacy, University of Marburg, Marburg, Germany

**Keywords:** Endocrine system and metabolic diseases, Ageing, Cardiovascular biology, Metabolism, Preclinical research, Cardiac hypertrophy, Cardiovascular diseases

## Abstract

Increased activation of sympathetic nervous system contributes to congestive heart failure (CHF) progression, and inhibition of sympathetic overactivation by beta-blockers is successful in CHF patients. Similarly, caloric restriction (CR) reduces sympathetic activity but mediates additional effects. Here, we compared the cardiac effects of CR (− 40% kcal, 3 months) with beta-blocker therapy (BB), diuretic medication (DF) or control diet in 18-months-old Wistar rats. We continuously recorded blood pressure, heart rate, body temperature and activity with telemetric devices and analysed cardiac function, activated signalling cascades and markers of apoptosis and mitochondrial biogenesis. During our study, left ventricular (LV) systolic function improved markedly (CR), mildly (BB) or even deteriorated (DF; control). Diastolic function was preserved by CR and BB but impaired by DF. CR reduced blood pressure identical to DF and BB and heart rate identical to BB. Plasma noradrenaline was decreased by CR and BB but increased by DF. Only CR reduced LV oxidative damage and apoptosis, induced AMPK and Akt phosphorylation and increased mitochondrial biogenesis. Thus, additive to the reduction of sympathetic activity, CR achieves protective effects on mitochondria and improves LV function and ROS damage in aged hearts. CR mechanisms may provide additional therapeutic targets compared to traditional CHF therapy.

## Introduction

Congestive heart failure (CHF) is a main contributor to mortality and reduction of quality of life in many patients. Whenever cardiac output begins to deteriorate, various neurohormones are activated to restore circulatory homeostasis. This neurohormonal activation in CHF, which involves increased activity of the sympathetic nervous system (SNS) and the renin-angiotensin aldosterone system (RAAS) among others, is initially a beneficial, adaptive response. However, excessive neurohormonal activation becomes maladaptive, leading to progression of CHF and poor long-term prognosis through a variety of mechanisms including loss of cardiomyocytes by apoptosis and necrosis, reactive hypertrophy of the remaining myocytes and increased fibrosis^1^. Likewise, the ageing heart is characterized by hyperactivation of various neurohormonal pathways, most importantly the SNS, and remodelling of the aged heart involves similar mechanism as described above for CHF. Although these mechanisms are not fully understood, the progressive accumulation of oxidative damage over lifetime has been recognized as a major contributing factor involved in cardiac ageing. Cardiomyocyte production of reactive oxygen species (ROS) occurs as a by-product of mitochondrial oxidative phosphorylation while other cellular sources such as NADPH oxidase and xanthine oxidase contribute less^2^. Ageing is associated with myocardial mitochondrial dysfunction, decreased mitochondrial biogenesis and impaired removal of dysfunctional mitochondria in the heart and increased oxidative stress^3^. Subsequently, oxidative damage to DNA, proteins or lipids accumulates with age and as a consequence, an impairment of cellular functions may occur.

Caloric restriction (CR) attenuates this age-associated increase in mitochondrial ROS production, delays signs of premature cardiac ageing and preserves mitochondrial function^4,5^. Lifelong CR has also been shown to improve the age-related impairments in diastolic function in mice^6^ as well as in Dahl salt-sensitive rats^7^. Furthermore, even when started late in life, CR remains cardioprotective in senescent myocardium by correcting pre-existing mitochondrial dysfunction and apoptotic activation and by preventing deterioration in left ventricular (LV) function^5^. The mechanisms underlying cardioprotective CR effects are diverse and among the known mediators for the benefits of CR are nitric oxide, the AMP-activated protein kinase (AMPK) and sirtuins. Activation of AMPK acts to maintain cellular energy stores and enhances oxidative metabolism and mitochondrial biogenesis. Previous studies suggest a strong activation of AMPK in the heart after long-term CR^5,8^ and after short-term CR in mice and rats^9^.

An increase in the activity of the SNS over the parasympathetic nervous system (PNS), as it occurs during ageing^10^, is a risk factor for cardiovascular diseases. Fasting or CR suppress sympathetic activity^11^ and reduce blood pressure in hypertensive and normotensive animals but also in humans^12^. Changes in SNS activation and reduction in blood pressure may represent major components of CR efficacy und were therefore compared with common blood pressure lowering drugs in the present study. Both, diuretics and beta-blockers, are used as antihypertensive drugs, but they differ concerning their influence on SNS activity. The short-acting loop diuretic furosemide decreases blood pressure but has also been described to increase sympathetic nerve activity^13^. Beta-blockers decrease sympathetic activity, although their individual potency differs^14,15^.

Here, we analysed if CR (− 40% calories, 3 months) exhibits additional addressable effects in the aged myocardium apart from SNS modulation (beta-blocker) and/or blood pressure reduction (beta-blocker, diuretic) regarding (I) hemodynamic parameters, ECG, activity and body temperature, (II) LV systolic and diastolic function, (III) changes in stress-activated signalling pathways, (IV) activation of AMPK and mitochondrial biogenesis and (V) markers of oxidative stress and apoptosis.

## Results

### General characteristics

None of the animals died during the course of the study. CR resulted in a significant decrease in LV weight, body weight (BW) and LV/BW or LV/tibia length compared to control and treatment groups (Suppl. Table 1). DF treatment resulted in highest values for all these parameters (Suppl. Table 1). CR significantly reduced blood glucose and insulin levels, but we did not observe any modulation of glucose metabolism by BB or DF (Table 1). Both, CR and BB, reduced leptin concentration but the most pronounced effects were observed in senescent CR rats (Table 1). Only CR strongly increased plasma adiponectin levels (Table 1).

**Table 1:** Serum/blood parameters at the end of the study.

	Co	CR	BB	DF
Glucose (mmol/l)	6.41 ± 0.08	5.79 ± 0.21*	6.45 ± 0.13^#^	6.54 ± 0.17^#^
Insulin (ng/ml)	1.20 ± 0.09	0.81 ± 0.13*	1.13 ± 0.24	0.98 ± 0.19
Leptin (pg/ml)	364 ± 21	160 ± 18**	220 ± 19*^,#^	396 ± 26^##^
Adiponectin (µg/ml)	5.49 ± 0.27	13.48 ± 1.01***	5.26 ± 0.53^###^	6.07 ± 0.33^##^
BNP (pg/ml)	66.51 ± 8.36	42.19 ± 9.14*	35.84 ± 8.39*	71.07 ± 9.61^#^
TBARS (µM)	34.65 ± 6.96	36.37 ± 5.52	30.62 ± 2.86	35.16 ± 3.39
Carbonyl (nmol/mg)	1.78 ± 0.23	1.54 ± 0.39	1.73 ± 0.32	1.82 ± 0.41
Adrenaline (pg/ml)	180 ± 15	171 ± 23	123 ± 29*	194 ± 34
Noradrenaline (pg/ml)	402 ± 37	326 ± 26*	286 ± 15**	496 ± 31*^,#^

Co control diet, CR caloric restriction, BB beta-blocker, DF furosemide.

*p < 0.05, **p < 0.01, ***p < 0.001 vs. respective control; ^#^*p* < 0.05, ^##^*p* < 0.01, ^###^*p* < 0.001 vs. CR. All data are mean ± SEM (n = 10 per group).

**Figure 1:**
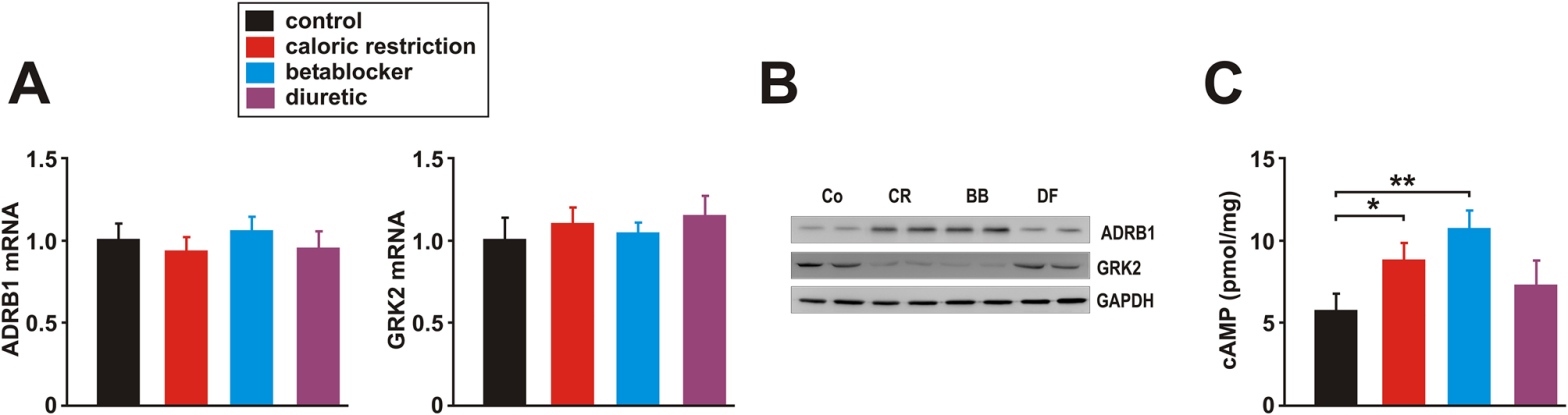
Effects of 3 months CR, beta-blocker or diuretic on beta1 adrenergic signalling. (**A**) Real-time PCR analyses of the relative copy number of beta1 adrenergic receptor (ADRB1) and GRK2 were performed in LV tissue (n = 10 per group). Threshold cycles (C_T_) of target genes were normalized to the mean of the housekeeping genes 18S rRNA, HPRT1 and GAPDH. (**B**) Representative Western blots of ADRB1 (membrane fraction) and GRK2 (whole cell lysate) in LV tissue (n = 10 per group). GAPDH served as loading control. (**C**) LV cAMP content was measured at the end of the study (n = 10 per group). *p < 0.05. All data are mean ± SEM. Co = control, CR = caloric restriction, BB = beta-blocker, DF = diuretic. Full-size blots are shown in Suppl. Fig. 2.

Baseline activity was significantly higher in all groups at night suggesting maintenance of circadian rhythm. Neither during periods of rest (POR; 7.00 a.m.–7.00 p.m.) nor periods of activity (POA; 7.00 p.m.–7.00 a.m.) mean activity differed between the groups (Suppl. Fig. 1A). Maximal activity during feeding was only slightly elevated in CR animals compared to the other groups (Suppl. Fig. 1A). Elevated peak activity in CR animals occurred mainly during episodes of re-feeding and POA but did not lead to an elevated overall-activity, suggesting the absence of additional physical activity effects in CR animals (Suppl. Fig. 1A). Body core temperature showed maintenance of circadian rhythm in all groups. Mean core temperature was mildly reduced by CR, DF and BB during POA compared to control animals and compared to the pre-treatment values (Suppl. Fig. 1B). Plasma adrenaline levels were moderately reduced by BB treatment while CR or DF did not modify them (Table 1). Plasma noradrenaline levels, however, were reduced by CR and BB, while DF treatment increased noradrenaline plasma levels significantly (Table 1). To get a deeper insight into the impact of these different treatments on adrenergic signalling in the heart, we investigated the expression of the beta1 adrenergic receptor (ADRB1) and of the G protein-coupled receptor kinase 2 (GRK2) together with LV cyclic adenosine monophosphate (cAMP) levels. As shown in Fig. 1, although ADRB1 and GRK2 mRNA (Fig. 1A) were not significantly altered, ADRB1 protein in cardiac membranes was significantly increased by BB and CR while GRK2 protein was significantly reduced by these two treatments compared to control rats (Fig. 1B). LV cAMP was highest in the BB group but also showed a significant increase in CR animals (Fig. 1C).

### Characterization of cardiac function and hemodynamics

Permanent central blood pressure was recorded by a telemetric transmitter (within the abdominal aorta throughout the study 24 h/day/3 months, n = 5 per group). Furthermore, all animals underwent standardized echocardiographic monitoring at the beginning, after 1.5 months and at the end of the study (n = 10 per group). Before organ harvesting, a close-chest-Millar-tip-catheterization was performed and aortic, central venous and the LV pressure and the rate in rise and fall of ventricular pressure were recorded continuously (n = 5 per group). The results of this deep cardiac characterization obtained throughout the study are presented in the following 4 subsections.

(a) LV systolic function.

**Figure 2:**
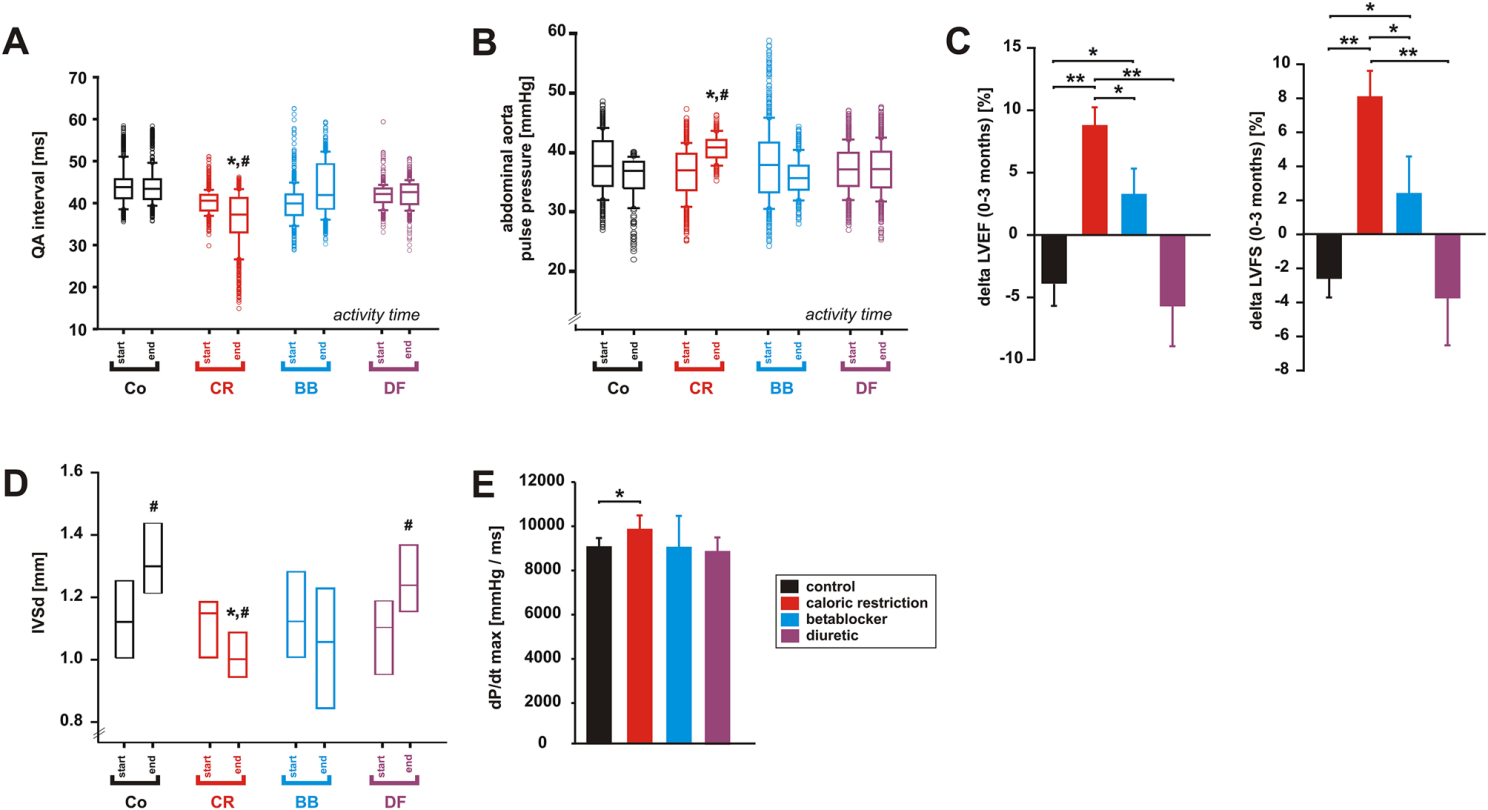
Effects of 3 months CR, beta-blocker or diuretic on LV systolic function. (**A**) Changes in QA interval and (**B**) in pulse pressure during the study (n = 5 per group). *p < 0.05 vs. control, ^#^*p* < 0.05 vs. respective value at the beginning of the study. (**C**) Changes in LV ejection fraction (ΔEF%) and in LV fractional shortening (ΔFS%) during the study (n = 10 per group). *p < 0.05; **p < 0.01. (**D**) Changes in interventricular septum diameter in diastole (IVSd) during the study. *p < 0.05 vs. control, ^#^*p* < 0.05 vs. respective value at the beginning of the study. (**E**) Maximal rate of rise of LV pressure (dP/dt max) at the end of the study (n = 5 per group). *p < 0.05. All data are mean ± SEM. Co = control, CR = caloric restriction, BB = beta-blocker, DF = diuretic. Data were obtained by telemetry (**A**, **B**), echocardiography (**C**, **D**) or catheter measurements (**E**).

A reduction of the QA interval (QAI), the interval between the Q wave and the onset of the aortic blood pressure pulse, is an indicator of increased cardiac contractility^16^. CR resulted in a significant decrease in QAI, while BB treatment mildly prolonged QAI during POA (Fig. 2A). DF or control diet did not modify QAI (Fig. 2A). Pulse pressure was significantly elevated by CR, mildly reduced by BB and maintained under DF treatment during POA (Fig. 2B). During POR, no significant difference was observed (not shown).

As animals were assigned to one of the four groups according to their LV function, the successful matching resulted in an equal distribution of LV ejection fraction (LVEF) and LV fractional shortening (LVFS) at the beginning of the study. In the course of the study (3 months) control animals experienced a mild deterioration in LV function (Fig. 2C). However, CR resulted in a 9.2 ± 1.5% increase in LVEF and in an 8.0 ± 1.6% increase in LVFS compared to animals receiving control diet during the study (Fig. 2C). BB rats showed only a mild improvement in these parameters and DF rats demonstrated a similar reduction in LVEF and LVFS compared to control rats (Fig. 2C). Furthermore, control and DF animals showed a significant increase of interventricular septum diameter in diastole (IVSd) suggesting moderate hypertrophy (Fig. 2D). BB did not modify IVSd, while CR induced a mild reduction in IVSd (Fig. 2D). Interventricular septum diameter in systole (IVSs) did not differ between the 4 groups (not shown). Millar-tip-catheterization revealed a mild increase in dp/dt max in CR animals, suggesting a moderately increased contractility, while no significant change was observed in BB or DF rats in comparison to controls (Fig. 2E). Ventricular diameters or the ratio of left atrial (LA) diameter to aortic diameter, identifying LA enlargement^17^, were not altered (not shown). Plasma BNP was significantly lower in CR and BB animals compared to control and DF animals (Table 1), although the observed maximal levels are significantly lower than those commonly observed in CHF rats^18^.

(b) LV diastolic function.

**Figure 3:**
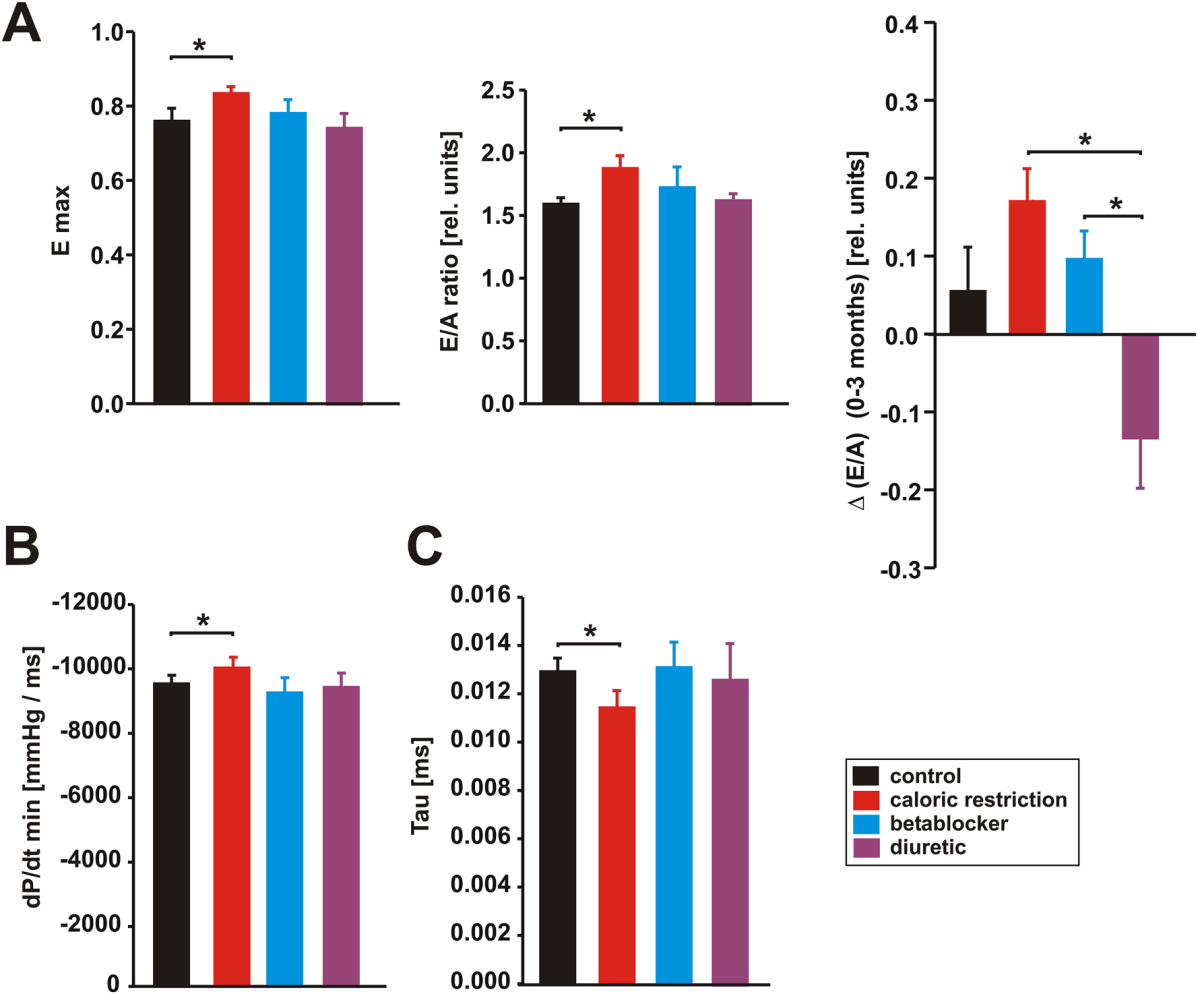
Effects of 3 months CR, beta-blocker or diuretic on LV diastolic function. (**A**) Maximal early inflow velocity (Emax, left panel), ratio of the early to late (**A**) ventricular filling velocities (E/A ratio, middle panel) at the end of the study and changes in E/A ratio (ΔE/A, right panel) during the study (n = 10 per group). *p < 0.05. (**B**) Maximal rate of pressure decrease (dt/dt min, left panel) and time constant of isovolumic LV pressure decay (Tau, right panel) at the end of the study (n = 5 per group). *p < 0.05. All data are mean ± SEM. Data were obtained by echocardiography (**A**), catheter measurements (**B**) or telemetry (**C**).

We analysed the transmitral flow velocity during early (E) and atrial (A) filling of the ventricle in order to obtain a marker of diastolic function and calculated the E/A ratio as a key measure showing abnormalities in LV filling during diastole. CR induced a mild increase in E max (Fig. 3A), while the A wave was not altered in any group compared to control rats (not shown). Thus, the E/A ratio increased mildly in CR animals compared to controls at the end of the study (Fig. 3A). Nonetheless, CR and BB resulted in maintenance of E/A in the course of the study, while DF resulted in a reduction of E/A compared to BB and CR treatment, reflecting nuanced effects of each intervention (Fig. 3A). Further characterization of diastolic function was performed by maximal relaxation, i.e. dP/dt min and the time constant of isovolumic LV pressure decay τ. CR resulted in an improved relaxation (dp/dt min, time constant τ), while BB and DF did not modify τ or dp/dt min compared to controls (Fig. 3B,C).

(c) Aortic arterial blood pressure and left ventricular pressures.

**Figure 4:**
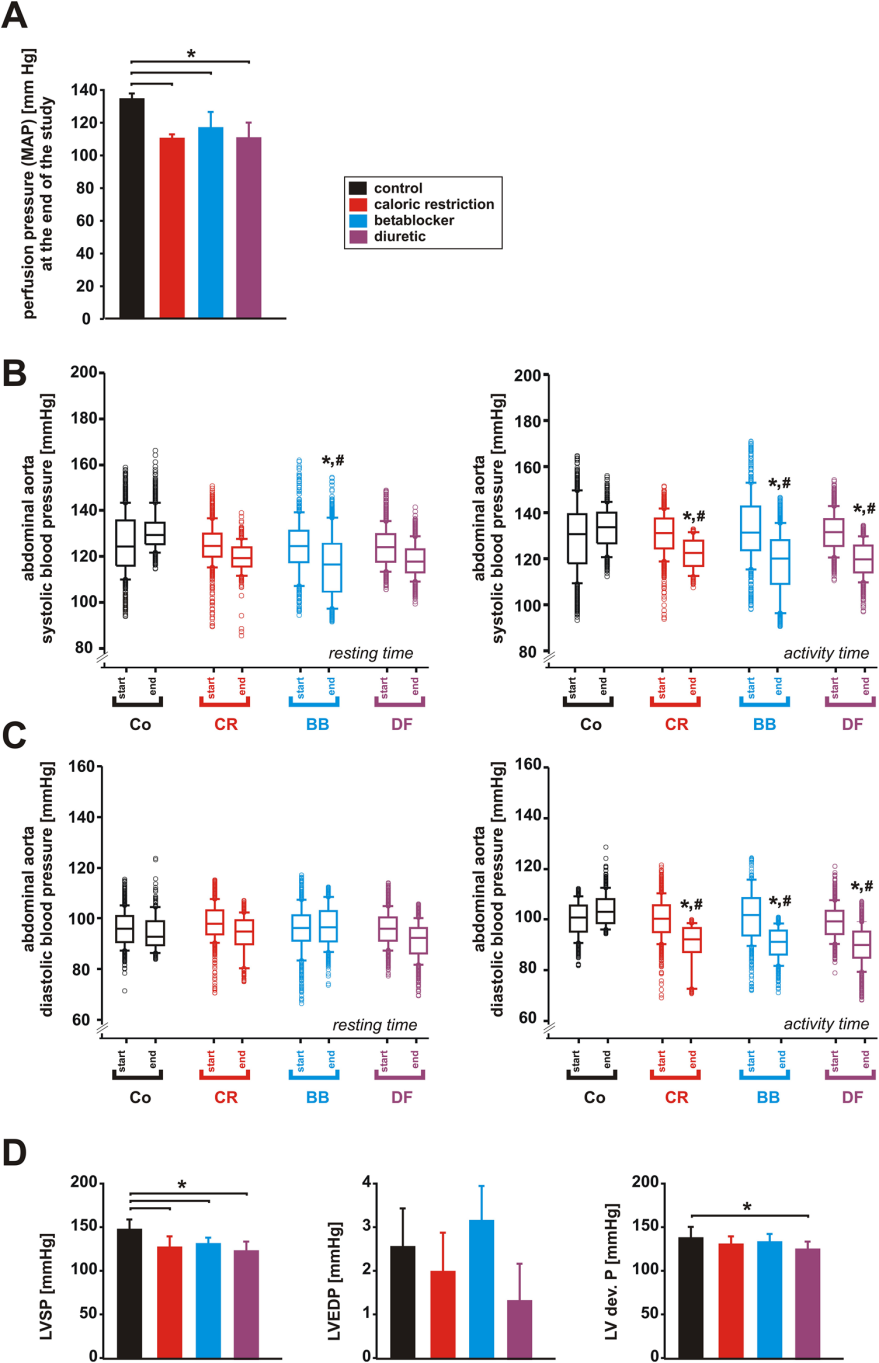
Effects of 3 months CR, beta-blocker or diuretic on arterial and LV pressure. (**A**) Mean arterial pressure at the end of the study (n = 5 per group). *p < 0.05. (**B**) Systolic and (**C**) diastolic blood pressure in the abdominal aorta during resting and activity time at the beginning and at the end of the study. *p < 0.05 vs. control, ^#^*p* < 0.05 vs. respective value at the beginning of the study (n = 5 per group). (**D**) LV systolic pressure (LVSP), LV enddiastolic pressure (LVEDP) and LV developed pressure (LV dev. P) at the end of the study (n = 5 per group). All data are mean ± SEM. Co = control, CR = caloric restriction, BB = beta-blocker, DF = diuretic. Data were obtained by catheter measurements (**A**), telemetry (**B**, **C**) or echocardiography (**D**).

Mean arterial blood pressure (MAP) as obtained by Millar tip measurement at the end of the study was similarly reduced by CR, BB and DF compared to controls (Fig. 4A). Overall effects obtained by continuous telemetric blood pressure measurement (24 h/day for 3 months) in the abdominal aorta were comparable during POR and POA but showed some variance regarding their impact on systolic and diastolic blood pressure. During POR only BB treatment induced a reduction in systolic pressure, while during POA CR, BB and DF significantly reduced systolic pressure compared to controls at the end of the study and compared to their respective values at the beginning of the study (Fig. 4B). Similarly, diastolic pressure was markedly reduced by CR, BB and DF during POA but not significantly modified during POR (Fig. 4C). Systolic left ventricular pressure (LVSP) was comparably reduced in all treatment groups (Fig. 4D). LVEDP varied mildly but not significantly between the groups (Fig. 4D). Maximal developed LV pressure (LVSP-LVEDP) was not modified by CR or BB but mildly reduced by DF compared to controls (Fig. 4D).

(d) Changes in heart rate spectrum.

**Figure 5:**
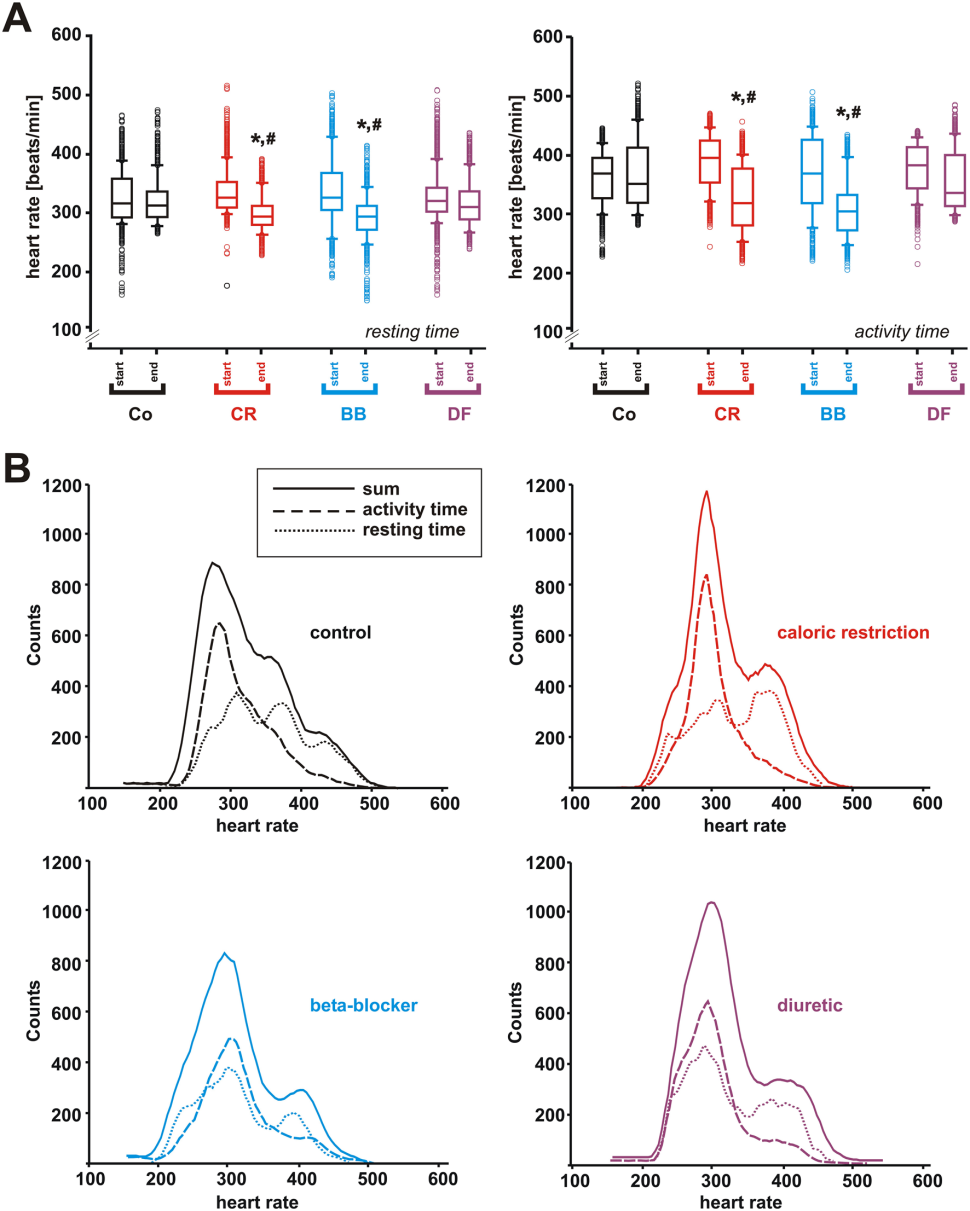
Effects of 3 months CR, beta-blocker or diuretic on heart rate spectrum. (**A**) Heart rate during resting (left panel) and activity time (right panel) at the beginning and at the end of the study. *p < 0.05 vs. control, ^#^*p* < 0.05 vs. respective value at the beginning of the study (n = 5 per group). (**B**) Heart rate distribution during activity time, resting time and the sum of both in all 4 treatment groups. All data are mean ± SEM. Co = control, CR = caloric restriction, BB = beta-blocker, DF = diuretic. All data were obtained by telemetry.

Heart rate (HR) variability (HRV) and chronotropic competence were maintained in all groups (Fig. 5A). Mean HR was significantly higher during POA compared to POR, and CR and BB reduced mean HR equally (Fig. 5A). POA were characterized by increased counts in high-frequency HRs compared to POR. Nevertheless, analysis of HR spectrum demonstrated reduced maximal HR and reduced periods of maximal HR during POA in CR and BB but not in DF animals compared to controls at the end of the study and compared to the respective values at the beginning of the study (Fig. 5B). During POR significantly reduced HR was achieved in BB animals but maximum effectiveness to reduce overall HR was accomplished by CR (Fig. 5B). Thus, CR and BB both reduced maximal HR without disturbing chronotropic competence. In addition, CR shows pronounced effectiveness during POA thus maintaining circadian variability.

### Signalling pathway activation and mitochondrial biogenesis

**Figure 6:**
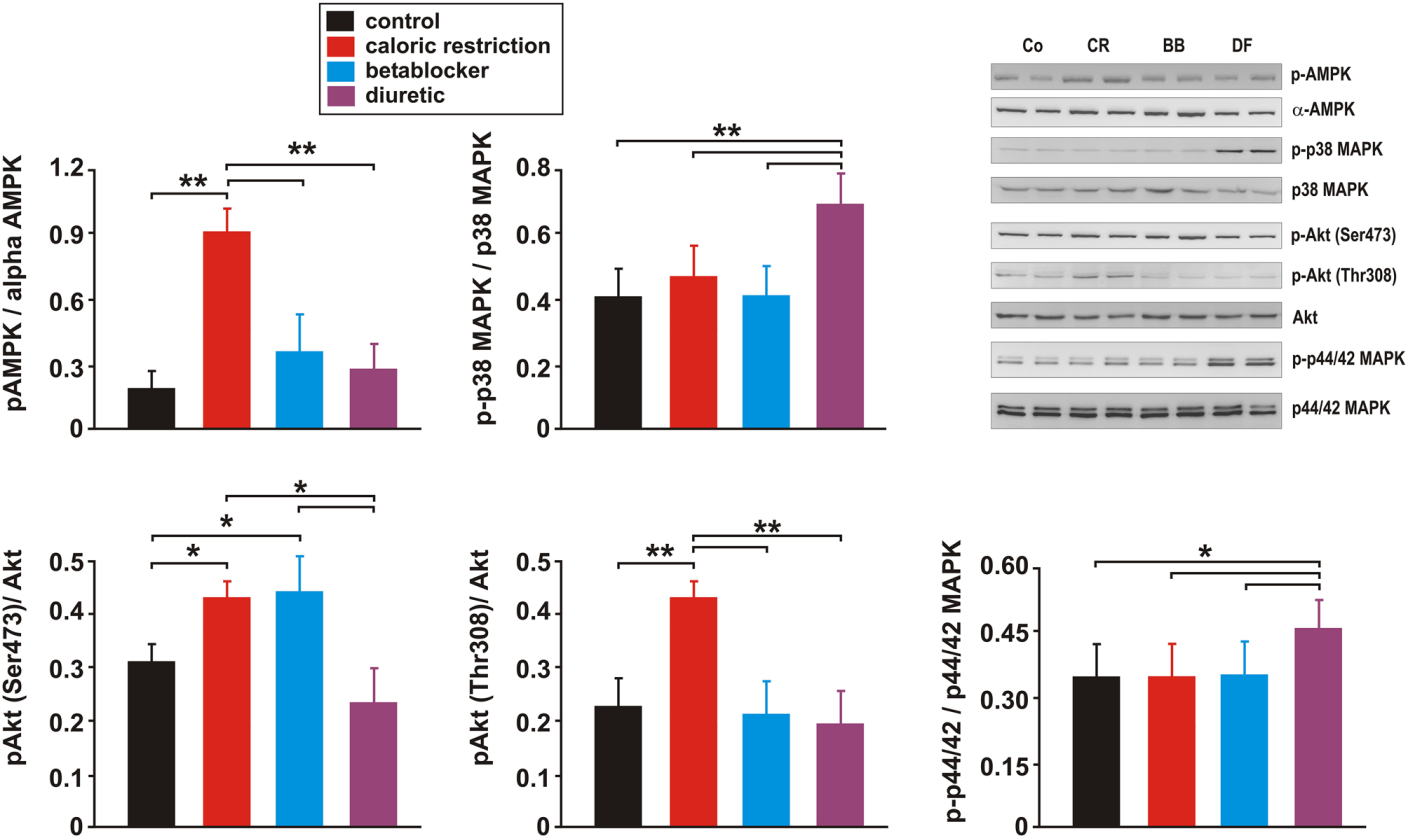
Effects of 3 months CR, beta-blocker or diuretic on LV signalling pathway activation. Representative Western blots and quantification of phosphorylation of AMPK (at Thr172), p44/42 MAPK (at Thr202/Tyr204), Akt (at Ser473 and at Thr308) and p38 MAPK (Thr180/Tyr182), each normalized to the respective total kinase, in LV tissue (n = 10 per group). *p < 0.05; **p < 0.01. All data are mean ± SEM. Co = control, CR = caloric restriction, BB = beta-blocker, DF = diuretic. Full-size blots are shown in Suppl. Fig. 3.

CR resulted in increased phosphorylation of AMPK at Thr172 and Akt at Ser473 and Thr308 while phosphorylation of the stress-activated p38MAPK and p44/42MAPK was not altered compared to controls (Fig. 6, Suppl. Fig. 3). BB increased phosphorylation of Akt only at Ser473 significantly but did not modify phosphorylation of AMPK, p38MAPK and p44/42MAPK (Fig. 6, Suppl. Fig. 3). DF treatment did not modify phosphorylation of AMPK or Akt but phosphorylation of the stress-activated p38MAPK and p44/42MAPK was increased by DF treatment compared to controls (Fig. 6, Suppl. Fig. 3).

**Figure 7:**
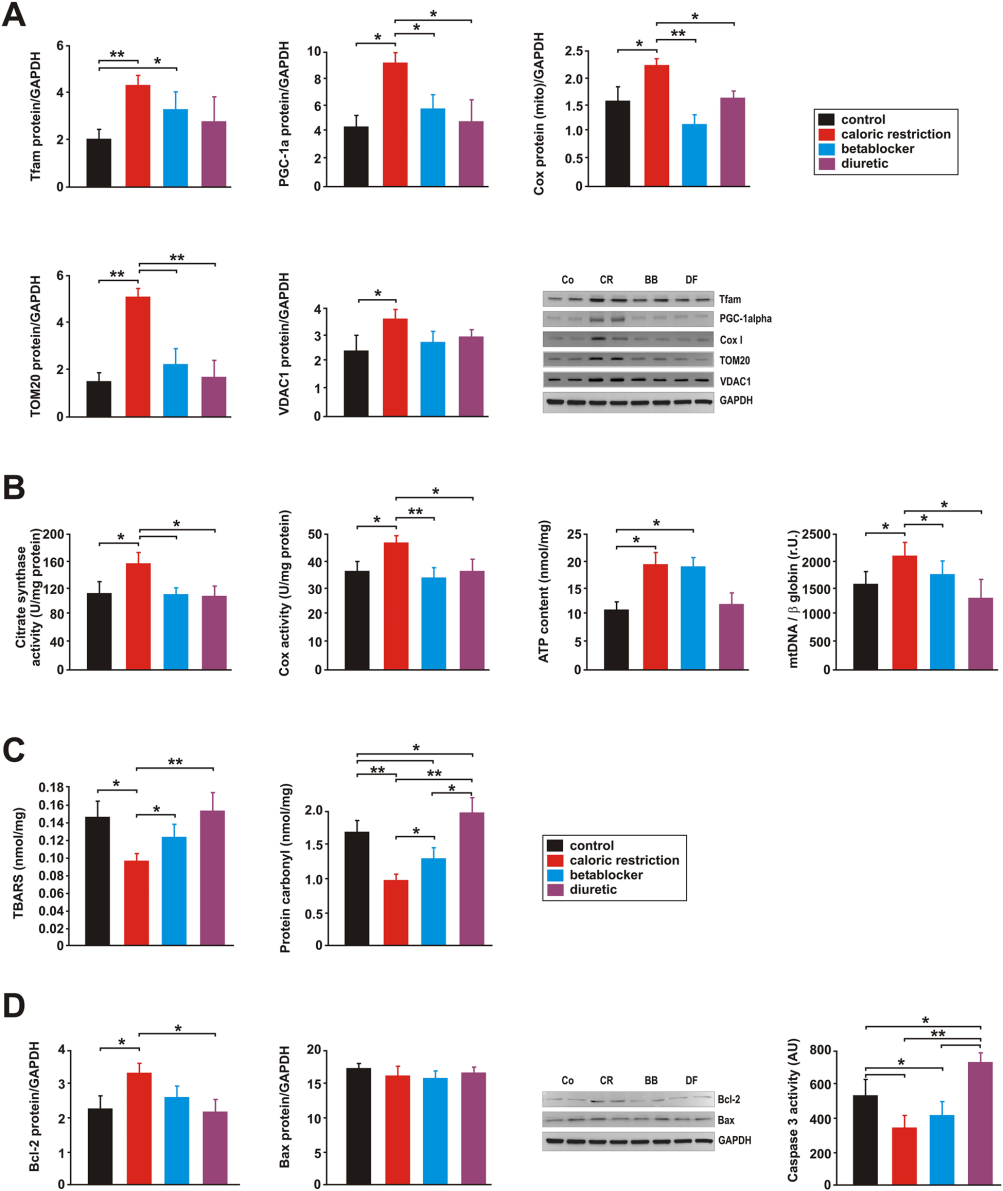
Effects of 3 months CR, beta-blocker or diuretic on LV mitochondrial biogenesis, oxidative stress and apoptosis. (**A**) Representative Western blots and quantification of protein expression of Tfam, PGC-1alpha, a mitochondrial component of complex IV (Cox I), TOM20 and VDAC1 in LV tissue (n = 10 per group). GAPDH served as loading control. *p < 0.05; **p < 0.01. (**B**) Citrate synthase enzyme activity (units/mg protein) as a marker of mitochondrial content, cytochrome c oxidase (COX) activity and ATP content were analysed in LV tissue lysates (n = 8 per group). In addition, real-time PCR measurements of the relative copy number of mtDNA per diploid nuclear genome (n = 10 per group) were performed. *p < 0.05; **p < 0.01. (**C**) Thiobarbituric Acid Reactive Substances (TBARS) and protein carbonyl content were measured in LV tissue at the end of the study (n = 10 per group). *p < 0.05; **p < 0.01. (**D**) Quantification and representative Western blots of Bcl-2 and Bax protein in LV tissue. GAPDH served as loading control. LV Caspase 3 activity was measured in tissue lysates (n = 10 per group) by a colorimetric assay and is given as artificial units (A.U.). *p < 0.05; **p < 0.01. All data are mean ± SEM. Co = control, CR = caloric restriction, BB = beta-blocker, DF = diuretic. Full-size blots are shown in Suppl. Fig. 5.

Both, AMPK and Akt promote mitochondrial biogenesis^19,20^. Indeed, CR consistently increased PGC-1alpha mRNA or protein expression and Tfam protein expression, as major regulators of mitochondrial biogenesis (Fig. 7A, Suppl. Figs. 4, 5). Accordingly, mRNA and protein expression of the primary mitochondrial transcript COX I increased in response to CR (Fig. 7A, Suppl. Figs. 4, 5). Furthermore, the levels of mitochondrial membrane proteins correlating with mitochondrial abundance such as translocase of outer membrane 20 (TOM20) and voltage-dependent anion channel 1 (VDAC1) were induced following CR but in none of the other groups (Fig. 7A, Suppl. Fig. 5). Similarly, citrate synthase and COX activity as well as ATP and mitochondrial DNA (mtDNA) content were significantly increased by CR compared to control animals (Fig. 7B).

Although BB resulted in a minor upregulation of Tfam protein expression compared to control rats, no increase in other markers of mitochondrial biogenesis was observed (Fig. 7A,B, Suppl. Figs. 4, 5). However, ATP content was also increased in BB animals, reaching similar levels as in CR animals (Fig. 7B). DF treatment did not alter any of the parameters related to mitochondrial biogenesis (Fig. 7A,B, Suppl. Figs. 4, 5).

### Oxidative damage, apoptotic and inflammatory parameters

The measured values for plasma thiobarbituric acid reactive substances (TBARS), a marker of lipid peroxidation, and for plasma protein carbonyl content, a marker of oxidative protein damage, were not different between the groups (Table 1). However, LV TBARS and LV protein carbonyl content were significantly decreased by CR, while they were mildly increased by DF (protein carbonyls) and mildly decreased by BB (TBARS) compared to the control group (Fig. 7C). Both parameters were significantly higher in the DF and BB group compared to the CR group (Fig. 7C). Oxidative stress has an impact on inflammatory signals and apoptotic cell death. Our analyses of changes in the mRNA expression of pro-inflammatory cytokines revealed a reduced tumor necrosis factors-alpha (TNF-alpha) and interleukin-1beta (IL-1beta) expression in the CR group compared to control, BB and DF animals but an increased expression of interleukin-6 (IL-6) and IL-1beta in the DF group compared to control and CR animals (Suppl. Fig. 6). Chronic CR also induced an increased protein expression of the anti-apoptotic protein Bcl-2 compared to control and DF animals while the pro-apoptotic member of the Bcl-2 family Bax was not altered. This was associated with a lower caspase 3 activity measured in LV lysates of CR animals, which was also observed in BB animals without alterations in Bcl-2 proteins (Fig. 7D, Suppl. Fig. 5). DF treatment did not alter Bcl-2 and Bax protein expression, but resulted in a significantly higher caspase 3 activation compared to the control, CR and BB group (Fig. 7D).

## Discussion

Blood pressure and neurohormonal activation increase with age and compromise cardiovascular function^21^. The present study shows that CR reduces heart rate and blood pressure, results in improved LV function in aged rat hearts and most likely normalizes increased SNS activity. Three months of CR also induce a strong, chronic activation of AMPK and Akt, associated with positive effects on mitochondrial biogenesis, ROS production and apoptosis. Unlike CR, beta-blocker treatment mediates smaller effects on LV function but strongest blood pressure effects, and induces a weaker Akt activation compared to CR but no AMPK activation or anti-inflammatory effects. This is associated with a reduced apoptotic activation and ROS damage but unchanged mitochondrial biogenesis in the BB group, suggesting that strong AMPK activation is needed for the mitochondrial biogenic response. Despite comparable effects on blood pressure, the diuretic furosemide does not induce comparable protective effects on LV function or mitochondrial biogenesis and increases SNS activity, inflammatory or caspase activation and ROS damage.

In addition to the suppression of oxidative stress^22,23^, CR also confers anti-inflammatory cellular effects as described before in animal models or humans^24^. Our study confirms an impact of CR on the expression of pro-inflammatory cytokines, which was not observed in response to metoprolol. The latter effect is unexpected since beta-blockade was shown to attenuate pro-inflammatory cytokines such as IL-1beta^25^, TNF-alpha^25^ or IL-6^26^ in postinfarct rats or reduce LV inflammation in aged spontaneously hypertensive rats^27^. Diuretic treatment on the other hand strongly increased the expression of pro-inflammatory cytokines in the LV, an effect which has not been described so far. Unlike the effects observed here in cardiac tissue, blood cells from CHF patients respond to furosemide with a decreased release of TNF-alpha and IL-6 in vitro^28^.

### Role of reduced SNS activation in mediating the observed CR effects

CHF represents a life-threatening morbidity with a severe reduction in quality of life and survival. It is associated with an autonomic imbalance, characterized by reduced parasympathetic activity and increased sympathetic activity, which plays a major role in the pathophysiology and progression of heart failure. Accordingly, well-established heart failure therapeutics such as beta-blockers or angiotensin-converting-enzyme (ACE) inhibitors target at suppressing sympathetic overactivation^1^. The efficacy of most beta-blockers in this process also includes normalization of the decreased ADRB1 expression, of the reduced cardiac cAMP levels and of the increased GRK2 expression^29,30^, effects observed in our study in response to BB as well as CR treatment. Interestingly, GRK2 and ADRB1 protein expression was altered in response to CR and BB despite unchanged mRNA levels (Fig. 1). The ADRB1 mRNA stability is known to be regulated via interaction of its 3′ untranslated region with the RNA binding proteins HuR or AUF1^31^. In addition, other mechanisms such as regulation of protein expression by noncoding RNAs may be involved. It is conceivable that these mechanisms may also be involved in the observed effects on ADRB1 and GRK2 expression in our animals.

There is an inverse relation between heart rate and life expectancy in mammals^32^, and an increase in heart rate is an independent risk factor in patients with or without cardiovascular disease^33^. Clinical trials suggest that heart rate reduction is an important beneficial mechanism of beta-blockers^33^. Although all beta-blockers decrease heart rate, there are significant differences in their cellular effects. In particular carvedilol, a non-selective beta-blocker with alpha1-blocker properties, mediates pleiotropic additional actions including anti-oxidant, anti-apoptotic, anti-proliferative, anti-ischemic and metabolic effects^34^. In addition, a strong increase in cardiac beta-receptor density has been observed with metoprolol therapy but not with carvedilol therapy^35^, suggesting that the ADRB1 upregulation observed with metoprolol would probably not have been observed with carvedilol. While metoprolol did not mediate effects on mitochondrial biogenesis, mitochondrial function or ROS damage in our study, carvedilol was shown to have a direct positive impact on cardiac mitochondria^36^. Thus, future studies should also compare the cardioprotection of CR and with carvedilol, a beta-blocker with strong anti-oxidative and mitochondrio-protective effects.

In young ischemic CHF animals, chronic CR mediates beneficial effects on cardiac function through modulation of adrenergic responsiveness and prevention of ADRB1 downregulation^37^. Similarly, our data (ADRB1 and GRK protein expression, catecholamine plasma levels, heart rate spectrum) suggest that normalization in SNS activity was achieved by CR, even when started at advanced aged and for a shorter interval as compared to other studies^37^. This is in accordance with previous human and animal studies, where weight loss improved SNS/PNS balance, demonstrated by increased HRV^38–40^.

### Role of adipokines in mediating the observed CR effects

CR strongly reduced the release of the adipokine leptin and increased the release of the adipokine adiponectin. Beta-blocker treatment on the other hand caused only a mild reduction on plasma leptin. Interestingly, leptin is not only involved in whole body energy metabolism but also increases overall sympathetic activity^41^. Accordingly, decreased leptin levels following weight loss are associated with decreased SNS activity, which is reversed after leptin administration^42^. Unlike CR, DF mildly increased plasma leptin, corresponding to its effect on SNS activity. Thus, reduced leptin levels may represent an important determinant of reduced SNS activity in CR animals. In addition, leptin was shown to be an important downstream factor mediating the hypertrophic effects of both angiotensin II and endothelin-1, which involves an increased p38MAPK and p44/42MAPK phosphorylation in cardiomyocytes^43^. In DF animals, high leptin levels were associated with activation of the p38MAPK and p44/42MAPK and a significant increase in IVSd during the course of the study suggesting hypertrophic growth. On the other hand, adiponectin, which is increased in CR animals, attenuates cardiomyocyte hypertrophy in response to various in vitro and in vivo stimuli, involving activation of AMPK and inhibition of p44/42MAPK signalling^44,45^.

### Role of AMPK activation in mediating the observed CR effects

Oxidative stress is a key contributing factor in the development of cardiac hypertrophy, which can be induced by angiotensin II, endothelin-1 as well as catecholamines. Neurohormonal overactivation in CHF thus contributes to accelerated ROS generation and deterioration of heart failure. Here, we demonstrate reduced ROS damage by CR, which may be related to AMPK. Indeed, AMPK activation leads to an increased transcription of various anti-oxidative enzymes including thioredoxin, MnSOD and catalase^46–48^. A mild reduction of ROS damage (only protein carbonyls) was also observed by us in the metoprolol group, although others have shown that carvedilol but not metoprolol mediates anti-oxidative effects^49^. The reduced ROS production may have contributed to the attenuated apoptotic activation as well as the inhibition of ROS-induced cardiomyocyte hypertrophy^44,45^ in BB and CR animals. Furthermore, impaired mitochondrial function has been described to lead to increased ROS release from mitochondria and subsequently to cardiomyocyte apoptosis^50,51^. On the other hand, carvedilol, a beta-blocker with anti-oxidant effects, or mitochondria-targeted agents such as the antioxidant MitoQ(10) mediate strong cardioprotective effects by preventing mitochondrial oxidative damage^36,52^.Thus, it can be envisioned that the reduction of ROS damage and the increased mitochondrial biogenesis, possibly associated with removal of dysfunctional mitochondria prone for ROS release, as observed in our study following CR may have contributed to reduced caspase 3 activity, suggesting reduced apoptotic cell death.

AMPK is also a major regulator of muscular mitochondrial biogenesis^19^, and it was shown that AMPK is necessary for normal mitochondrial function in the heart^53^. Among others, AMPK is activated by the adipokine adiponectin^54^. Our study suggests an activation of mitochondrial biogenesis exclusively by CR which was associated with a strong increase in plasma adiponectin and LV AMPK activation. These changes were not observed in the BB or DF group. Although RAAS inhibition by valsartan^55^ was shown to increase mitochondrial biogenesis in experimental hypertension, no comparable data exist for the effects of beta-blockers on cardiac mitochondrial biogenesis. An activation of Akt, which plays a critical role in regulating growth, proliferation and survival, was observed in response to CR and BB. This was associated with a reduced apoptotic activation and ROS damage but unchanged mitochondrial biogenesis, suggesting that AMPK activation is needed for the mitochondrial biogenic response.

### Study limitations

Although our study demonstrates an improved LV function and mitochondrial biogenesis in addition to effects also induced by beta-blockers through reduction of sympathetic activity in aged but otherwise healthy animals without CHF signs, CR represents a multifactorial intervention potentially harmful in patients or animals with CHF accompanied by cardiac cachexia. Furthermore, the application of a CR even more severe than in our study may mediate direct negative effects even in healthy individuals. Indeed, a 60% reduction in calories was recently shown to cause endothelial dysfunction and cardiac arrhythmia in rats^56^. Furthermore, severe CR was also reported to result in an increased activation of central RAAS in order to maintain blood pressure allostasis^57^ and in myocardial dysfunction due to impaired cardiac calcium handling in rats^58^. There are no controlled trials on the effects of long-term CR (comparable to the relative ratio between CR duration and lifetime as in animals) on cardiac function in senescent humans for obvious reasons including unresolved safety issues or difficulties in lifelong observation of participants. Furthermore, the efficacy of CR in humans remains controversial. However, similar to the data obtained from animal studies, biomarkers of longevity such as fasting insulin level or body temperature are decreased^59^ and major risk factors for coronary heart disease are substantially improved in humans after CR^60–62^. Besides modulating cardiovascular risk profiles, direct myocardial CR effects might offer additional targets to prevent age-associated cardiac dysfunction^63^. Thus, selective targeting of mechanisms activated by CR might be a therapeutic option in addition to currently established therapies targeting neurohormonal activation in CHF patients.

## Methods

### Animal model

Male Wistar rats (18 months, Charles River (Germany)) were caged individually with a light/dark cycle of 12 h and assigned to one of the 4 groups (n = 10 per group) as matched pairs (baseline LV function). Prior to application of diet protocols, daily food and water intake was individually monitored (14 days) and averaged. Rats were randomly assigned to one of the following diets for the next three months: control diet (Co): individual prediet average of standard chow Altromin 1244 (2550 cal/g); caloric restriction (CR): prediet average of a calorically reduced, fibre-rich diet (Altromin 1344/1500; 1550 cal/g); beta-blocker (BB) : control diet and tap water individually substituted with metoprolol succinate (250 mg/kg BW/day); diuretic treatment (DF): control diet and tap water individually substituted with furosemide (15 mg/kg BW/day). Pilot experiments had shown a blood pressure lowering effect comparable to chronic CR for these dosages of metoprolol or furosemide. Animals received a telemetric device as described below. All animal experiments were performed according to the regional authorities and ethics committees for animal research of the Martin-Luther-University Halle-Wittenberg and complied with the directive 2010/63/EU of the European Parliament on the protection of animals used for scientific purposes. The experiments were registered under the numbers 2-677-MLU and 2-785-MLU.

### Surgical implantation of transmitters

Fully ventilated rats (17 months) underwent implantation of an abdominal telemetry device (PhysioTel Model TL11M2-C50-PXT, Data Sciences International) for permanent monitoring of intra-aortic blood pressure, ECG, core-body temperature and activity under anaesthesia with 2% isoflurane and buprenorphine. An intra-aortal catheter was placed proximal of the renal arteries, transducers were packed in the abdominal omentum and fixed. Subcutaneous ECG electrodes were placed according to ECG lead II. Data were recorded continuously using Dataquest A.R.T.-Acquisition and Analysis Software (V 4.20 Transoma Medical, Data Sciences International) from conscious, freely moving, and individually caged animals. Rats were allowed to recover for 1 month before experimental diets.

### Echocardiography

Two-dimensional and M-mode echocardiographic examinations in rats anesthetized with isoflurane at concentrations of 1.5% was performed at the beginning, after 1.5 months and at end of study. These examinations were performed in accordance with the criteria of the American Society of Echocardiography using a 12.5-MHz probe (Vivid 5, GE Healthcare) by the same trained, blinded sonographer, evaluating both cardiac geometry and function.

### Invasive hemodynamic measurements

At the end of the study, cardiac function was measured in anesthetized, ventilated rats (buprenorphine, 2% isoflurane) with a 3F ultraminiature catheter (Millar Instruments Inc.), placed via the right carotid artery in the ascending aorta and in the left ventricle. Central venous pressure was measured accordingly by catheterization of the left jugular vein. HR, aortic pressure, central venous pressure and the LV pressure and the rate in rise and fall of ventricular pressure were recorded continuously using the acquisition software LabChart v7.3.8 (ADInstruments) for a period of 20 min after stabilization of catheter placement.

### RNA isolation, RT-PCR and qPCR

RNA was isolated from LV tissue with the RNeasy Mini Kit (Qiagen) according to the manufacturer’s instructions as described previously by us^64^. In brief, prior to cDNA synthesis, integrity and quality of the RNA was confirmed by gel electrophoresis and the concentration determined by measuring UV-absorption. Reverse transcription of RNA samples (500 ng total RNA) was carried out for 30 min at 42 °C using the SuperScript III First-Strand cDNA Synthesis Kit (ThermoFisher Scientific). Real-time PCR (Primer sequences: Supplementary Table 2) and data analysis were performed using the Mx3000P Multiplex Quantitative PCR System (Stratagene) as described^64^. Each assay was performed in duplicate and validation of the PCR runs was assessed by evaluation of the melting curve of the PCR products (primer sequences in Suppl. Table 2). Every reaction was performed as duplicates and quantified with the ΔΔC_T_-method. Threshold cycles (C_T_) of target genes were normalized to the mean of the housekeeping genes 18S rRNA, HPRT1 and GAPDH. The resulting ΔC_T_ values were compared to untreated control cells or young mice receiving control diet and relative mRNA expression was calculated by R = 2^−ΔΔCT^. Relative copy numbers of mtDNA per diploid nuclear genome were measured using a fragment of mtDNA (16S rRNA) and a fragment of beta-globin.

### Protein extraction and Western blotting

For the isolation of whole cell lysates, frozen LV tissue was rapidly homogenized in a buffer containing 50 mM Tris HCl, 150 mM NaCl, 5 mM EDTA, 0.1% SDS, 1% sodiumdeoxycholate and protease inhibitor cocktail (Sigma-Aldrich). Isolation of the membrane fraction was performed with the Mem-PER Plus Membrane Protein Extraction Kit (ThermoFisher Scientific). Proteins were quantified using the BCA Protein Assay (Pierce). 50 μg of protein were loaded on 10% or 12.5% SDS-PAGE gels. After electrophoresis, proteins were transferred to nitrocellulose membranes. Filters were blocked and incubated with antibodies against cytochrome oxidase I (COX I; ThermoFisher Scientific), Tfam (Santa Cruz), PGC-1alpha (EMD Millipore), Bcl-2, Bax, phospho-AMPK (Thr172), alpha-AMPK, phospho-p44/42 MAPK, p44/42 MAPK, phospho-Akt (Thr308 or Ser473), Akt, phospho-p38MAPK, p38MAPK, TOM20, VDAC1, GRK2 (all Cell Signaling), ADRB1 (Abcam) and GAPDH (Abcam). After incubation with a peroxidase-conjugated secondary antibody (1:10.000), blots were subjected to the enhanced chemiluminescent detection method with the Fusion FX7 imaging system (Peqlab).

### Mitochondrial enzyme activity

For the determination of mitochondrial enzyme activity, small pieces of frozen tissue were homogenized (1/30 weight per volume) in a solution containing 50 mM of Tris buffer (pH 7.5), 100 mM potassium chloride, 5 mM MgCl_2_, and 1 mM EDTA using a glass/glass homogenizer (Kontes Glass Co., Vineland, New Jersey, 2 ml, 0.025 mm clearance). Enzyme activities were assayed at 30 °C spectrophotometrically using a Cary 50 spectrophotometer (Varian Instruments) and related to total protein content in the same sample. Assays were run in duplicates with two different quantities of sample. Cytochrome c oxidase (COX) was measured by recording the decrease in absorbance during the oxidation of reduced cytochrome c at 550 nm. Citrate synthase was determined with DTNB, oxaloacetate, acetyl-CoA and 0.1% Triton X‐100 at 412 nm as described previously^65^.

### ATP content

ATP extraction from LV tissues was performed according to the protocol supplied by the manufacturer (ATP Colorimetric/Fluorometric Assay Kit, Sigma-Aldrich). LV tissue was removed from liquid nitrogen and immediately homogenized in 10 volumes of ATP assay buffer, followed by deproteinization with a 10 kDa Molecular Weight Cut-Off column (Abcam). After incubation with reaction mix for 30 min, the fluorescence was measured (excitation 535 nm, emission 587 nm) on a Fluostar Optima microplate fluorometer (BMG Laboratories). An aliquot of non-deproteinized sample was utilized for determination of protein content using the BCA Protein Assay (Pierce).

### Cyclic AMP content

The Direct cAMP ELISA kit (Enzo Life Sciences GmbH) was utilized according to the protocol supplied by the manufacturer. LV tissue was removed from liquid nitrogen and immediately homogenized in 10 volumes of 0.1 M HCl. After neutralization and incubation with cAMP antibody, cAMP conjugated to alkaline phosphatase and alkaline phosphatase substrate, the absorbance was measured at 405 nm. Protein content of tissue lysates was determined using the BCA Protein Assay (Pierce).

### Plasma analyses

Plasma BNP, adiponectin and leptin concentrations were measured by Enzyme-Linked-Immunosorbent-Assays (Rat BNP ELISA-kit, Assay Pro; Mouse/Rat Adiponectin ELISA-kit, B-Bridge International Inc.; Mouse/Rat Leptin ELISA-kit, BioVendor). Plasma insulin concentration was determined by radioimmunoassay (LINCO Research Inc.). Glucose concentrations were measured with the Glucose assay kit (BioCat, Heidelberg, Germany). Plasma adrenaline and noradrenaline were analysed by high performance liquid chromatography.

### Caspase 3 assay

10 mg LV tissue were homogenized in lysis buffer containing 50 mM HEPES, pH 7.4, 5 mM CHAPS, 5 mM DTT. Caspase 3 activity was measured with the Colorimetric Caspase 3 Assay Kit (Sigma-Aldrich) which is based on the hydrolysis of the peptide substrate acetyl-Asp-Glu-Val-Asp p-nitroanilide (Ac-DEVD-pNA) by caspase 3, resulting in the release of the p-nitroaniline, measured spectrophotometrically at 405 nm.

### Measurement of oxidative protein and lipid damage

Plasma or LV protein carbonyl content was measured with the Protein Carbonyl Assay Kit (Cayman Chemical) according to the protocol supplied by the manufacturer. Serum samples were utilized directly and tissue samples were homogenized in cold buffer containing 50 mM MES, pH 6.7 and 1 mM EDTA. The reaction between 2,4 dinitrophenylhydrazine (DNPH) and proteins, forming a Schiff base to produce the corresponding hydrazone, was utilized to estimate carbonyl content by measuring the absorbance spectrophotometrically at 370 nm. Thiobarbituric Acid Reactive Substances (TBARS) were measured with the TBARS assay Kit (Cayman Chemical). Serum samples were utilized directly and tissue was homogenized in RIPA buffer (10 mM Tris–HCl, pH 8, 140 mM NaCl, 1 mM EDTA, 1 mM EGTA, 1% Triton X-100). The adduct of malondialdehyde (MDA) and TBA formed under high temperature and acidic conditions was measured fluorometrically (excitation 530 nm, emission 550 nm) on a Fluostar Optima microplate fluorometer (BMG Laboratories). The protein content of the samples was determined using the BCA Protein Assay (Pierce).

### Statistical analysis

Numeric parameters are expressed as mean ± SEM unless otherwise stated. Data were analysed for normal distribution (Shapiro–Wilk test) and variance (Levene test) and subsequently analysed using 1-way analysis of variance (all pairwise multiple comparison procedure; Holm Sidack’s test; telemetric parameters: Dunn’s Method for correction; 4 groups, equal sample numbers). Targets were specified a priori. Statistical significance was accepted at *p* < 0.05. We used SigmaStat for Windows version 3.5 (Systat Software Inc.).

## Supplementary Information

Supplementary Information.

## Data Availability

The datasets generated and analysed during the current study are available from the corresponding author on request.
