# Supplementary material for: Caloric restriction reduces sympathetic activity similar to beta-blockers but conveys additional mitochondrio-protective effects in aged myocardium
Source: Sci Rep. 2021 Jan 21;11:1931. doi: 10.1038/s41598-021-81438-7 (PMC7820280; doi:10.1038/s41598-021-81438-7)
Supplement: Supplementary file 1 — Supplementary Information. [file 41598_2021_81438_MOESM1_ESM.pdf]

Caloric restriction reduces sympathetic activity similar to beta-blockers but conveys additional mitochondrio-protective effects in aged myocardium

Bernd Niemann, MD [1,3], Ling Li, MD [2], Andreas Simm, PhD [3,4],

Nicole Molenda, MS [2], Jens Kockskämper PhD [5], Andreas Boening, MD [1]

and Susanne Rohrbach, MD [2,*]

[1] Department of Cardiac and Vascular Surgery, Justus Liebig University Giessen and University Hospital Giessen and Marburg,

[2] Institute of Physiology, Justus Liebig University Giessen and

[3] Department of Cardiac Surgery and [4] Centre of Medical Basic Research, Martin Luther University Halle-Wittenberg

[5] Institute of Pharmacology and Clinical Pharmacy, University of Marburg

[*] Corresponding author:
Susanne Rohrbach, M.D.
Institute for Physiology
Justus Liebig University Giessen
Aulweg 129
35392 Giessen
Germany
susanne.rohrbach@physiologie.med.uni-giessen.de
Phone: 0049-641-9947268
Fax: 0049-641-9947269

Supplementary Table 1: Effects of caloric restriction on LV and body weight

	**Co**	**CR**	**BB**	**DF**
Left ventricle (g)	1.66± 0.06	1.19± 0.11 *	1.55± 0.06 #	1.74± 0.07 #
Body weight (g)	528±17	435±16 *	516±22 #	533±21 #
LV/BW (g/kg)	3.14±0.05	2.75±0.07 *	3.03±0.09	3.27±0.09 #
LV/tibia length (mg/cm)	33.9±0.9	24.8±0.6 **	31.7±0.4	35.4±0.8 ##

Co control diet, CR caloric restriction, BB beta-blocker, DF furosemide. *: p<0.05; **: p<0.01 vs. respective control; #: p<0.05; ##: p<0.01 vs. CR. All data are mean±SEM (n=10 per group).

Supplementary Table 2: Primer sequences

Gene	GenBank #	Forward Primer	Reverse Primer
Tfam	NM_031326.1	ATG GGC TTA GAG AAG GAA GCC	GTG ACT CAT CCT TAG CCC CC
PGC-1alpha	NM_031347	CCG AGA ATT CAT GGA GCA AT	GTG TGA GGA GGG TCA TCG TT
COXI	KM577634.1	GAT TCT TCG GAC ACC CAG AA	AGG CTC GCG TGT CTA CAT CT
beta-globin	NM_001113223.1	GCC TGT GGG GAA AGG TGA ATG	CTT CAC CTG GGG GTT ACC CAT
mtDNA (16S rRNA)	KM577634.1	AGT GAA GGG GCG GAC TCA TA	GAG GTC ACC CCA ACC GAA AT
IL-1beta	NM_031512.2	CTA TGT CTT GCC CGT GGA GC	CGT CAT CAT CCC ACG AGT CA
IL-6	NM_012589.2	CAC AAG TCC GGA GAG GAG AC	TCT GAC AGT GCA TCA TCG CT
TNF-alpha	NM_012675.3	TGA TCC GAG ATG TGG AAC TGG	CGA TCA CCC CGA AGT TCA GTA
ADRB1	NM_012701.1	GGC GCT CAT CGT GCT GCT CA	AGG CAC CAC CAG CAG TCC CA
GRK2	NM_012776.1	AAA GCC CCT TCT TCC GTT CC	GTT GCG GTA CAG TTC CTG GT
18S rRNA	NR_046237	TGG AGC GAT TTG TCT GGT TA	ACG CCA CTT GTC CCT CTA AG
GAPDH	NM_017008.4	CAC CAT CTT CCA GGA GCG AG	GAA GGG GCG GAG ATG ATG AC
HPRT-1	NM_012583.2	ACC AGT CAA CGG GGG ACA TA	ATT TTG GGG CTG TAC TGC TTG A

Supplementary Figures:

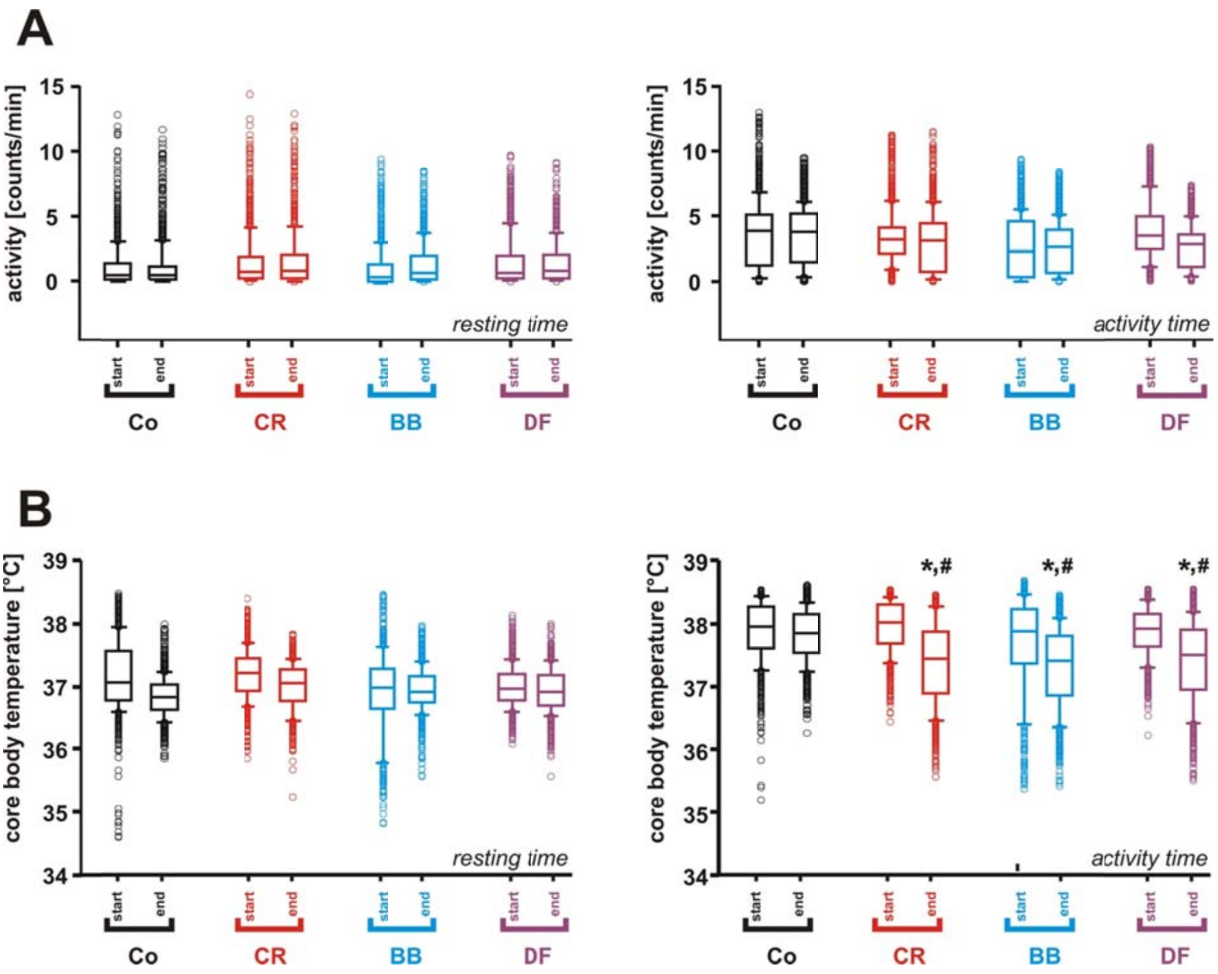

Supplementary Figure 1: Effects of 3 months CR, beta-blocker or diuretic on activity and body temperature

A: Activity during resting (left panel) and activity time (right panel) at the beginning and at the end of the study (n=5 per group).

B: Core body temperature during resting (left panel) and activity time (right panel) at the beginning and at the end of the study (n=5 per group).

All data are mean±SEM. Co=control, CR=caloric restriction, BB=beta-blocker, DF=diuretic.

*: $p<0.05$ vs. control, #: $p<0.05$ vs. respective value at the beginning of the study.

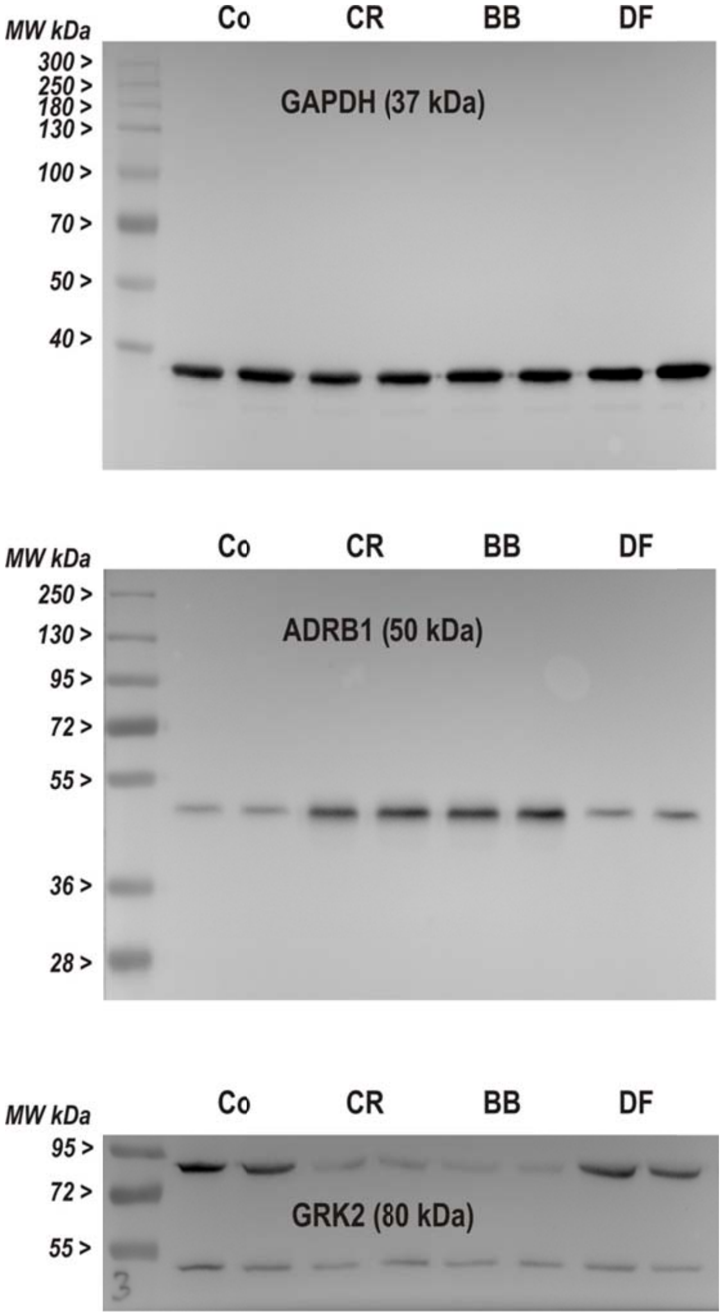

Supplementary Figure 2: Effects of 3 months CR, beta-blocker or diuretic on beta1 adrenergic signalling

Representative full-size Western blots of ADRB1, GRK2 and GAPDH in LV tissue. The merged pictures also include an according size marker. Images were taken with the Fusion FX7 imaging system and adjusted with Corel Photo-Paint.

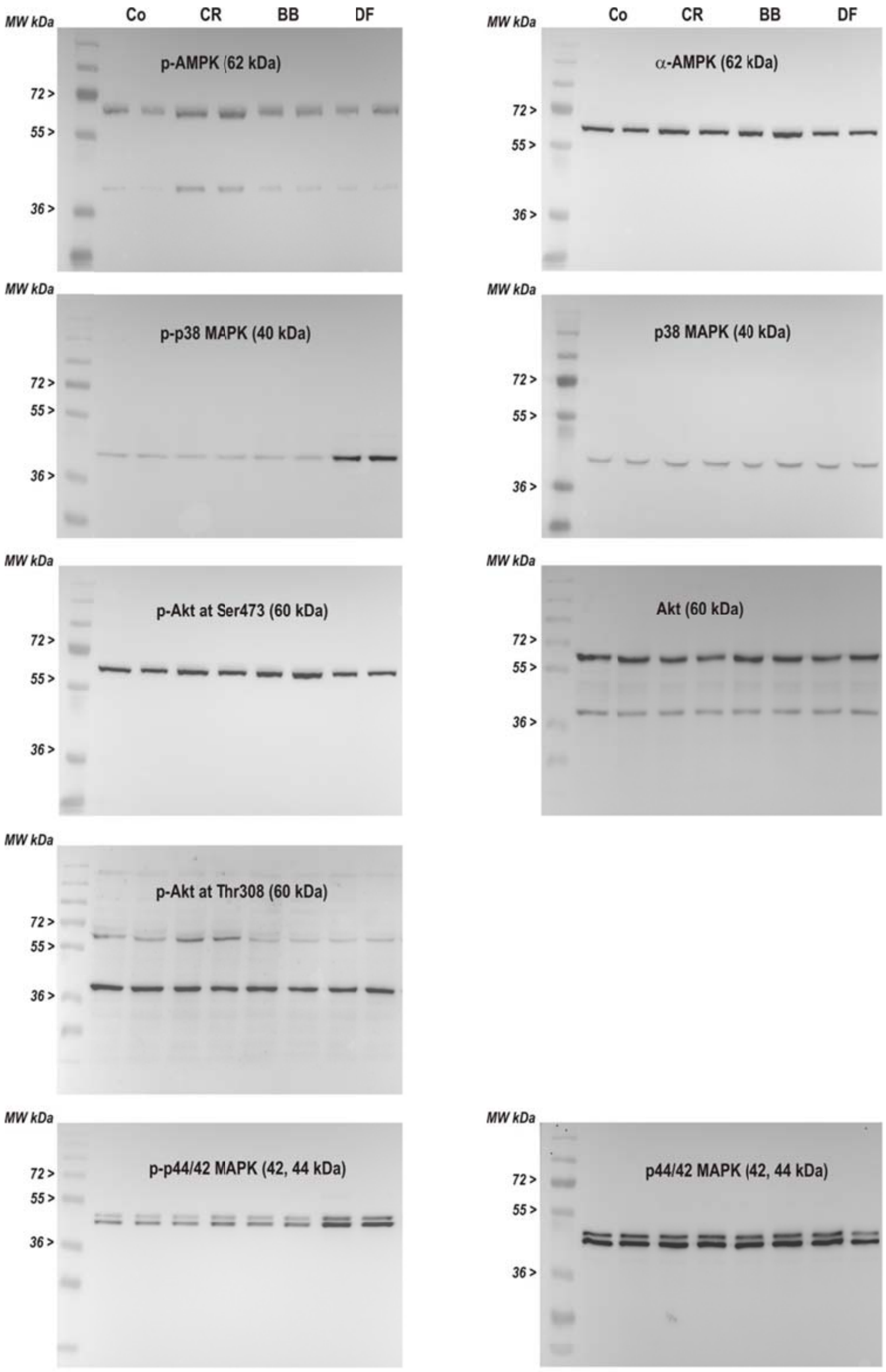

Supplementary Figure 3: Effects of 3 months CR, beta-blocker or diuretic on LV signalling pathway activation

Representative full-size Western blots of phospho-AMPK (at Thr172), total-AMPK, phospho-p38 MAPK (Thr180/Tyr182), total-p38 MAPK, phospho-Akt (at Ser473 and at Thr308), total-Akt, phospho-p44/42 MAPK (at Thr202/Tyr204) and total- p44/42 MAPK. The merged pictures also include an according size marker.

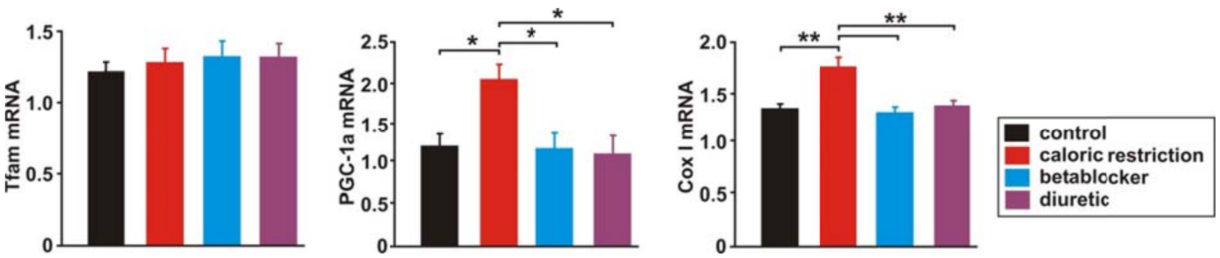

Supplementary Figure 4: Effects of 3 months CR, beta-blocker or diuretic on LV mitochondrial biogenesis

Real-time PCR analyses of the relative copy number of Tfam, PGC-1alpha and the primary mitochondrial transcript Cox I (complex IV) were performed in LV tissue (n=10 per group). Threshold cycles (C_T) of target genes were normalized to the mean of the housekeeping genes 18S rRNA, HPRT1 and GAPDH. All data are mean±SEM. *: $p<0.05$; **: $p<0.01$ vs. control.

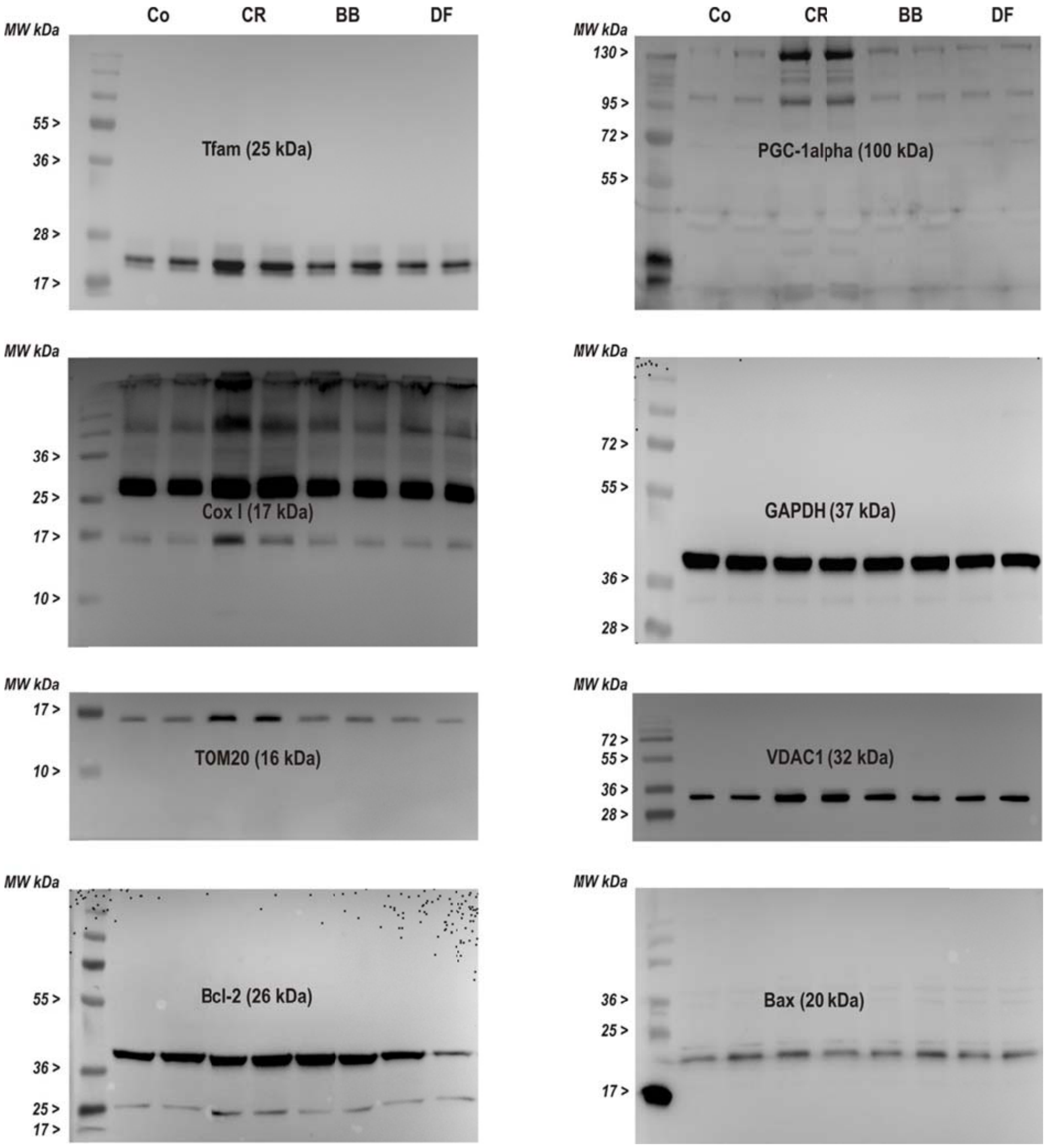

Supplementary Figure 5: Effects of 3 months CR, beta-blocker or diuretic on LV mitochondrial biogenesis and apoptosis

Representative full-size Western blots of Tfam, PGC-1alpha and a mitochondrial component of complex IV (Cox I), TOM20, VDAC1, Bcl-2 and Bax in LV tissue (n=10 per group). GAPDH served as loading control. The merged pictures also include an according size marker.

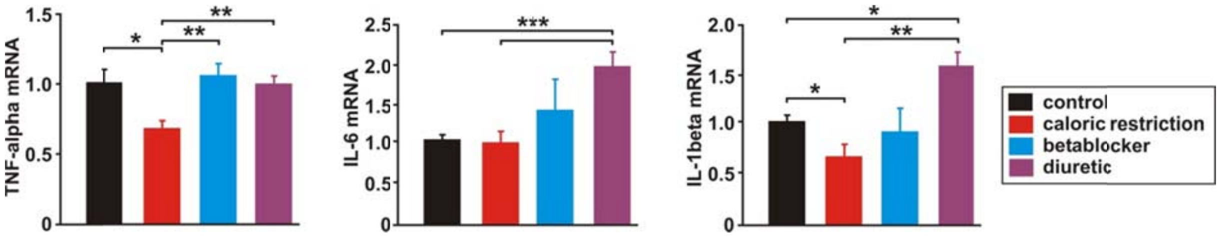

Supplementary Figure 6: Effects of 3 months CR, beta-blocker or diuretic on the expression of pro-inflammatory cytokines in the LV

Real-time PCR analyses of the relative copy number of TNF-alpha, IL-6 and IL-1beta were performed in LV tissue (n=10 per group). Threshold cycles (C_T) of target genes were normalized to the mean of the housekeeping genes 18S rRNA, HPRT1 and GAPDH. All data are mean±SEM. *: $p<0.05$; **: $p<0.01$; ***: $p<0.001$ vs. control.

List of abbreviations

ACE	angiotensin converting enzyme
ADRB1	adrenergic receptor beta 1
AMPK	AMP-activated protein kinase
BNP	brain natriuretic peptide
cAMP	cyclic adenosine monophosphate
CHF	congestive heart failure
CR	caloric restriction
GRK2	G protein-coupled receptor kinase 2
HR	heart rate
HRV	heart rate variability
IVSd	interventricular septal thickness at end-diastole
LA	left atrial
LV	left ventricle / left ventricular
LVEF	LV ejection fraction
LVFS	LV fractional shortening
LVSP	LV systolic pressure
MAPK	mitogen-activated protein kinase
PGC-1alpha	peroxisome proliferator-activated receptor gamma coactivator 1-alpha
PNS	peripheral nervous system
POA	period of activity
POR	period of resting
RAAS	renin angiotensin aldosterone system
ROS	reactive oxygen species
SNS	sympathetic nervous system
TBARS	thiobarbituric acid reactive substance
